# Effect of cervical Bishop score on induction of labor at term in primiparas using Foley catheter balloon: a retrospective study

**DOI:** 10.1186/s12884-024-06600-1

**Published:** 2024-05-31

**Authors:** Shu-Fen Li, Hui-Hui Ju, Chuan-Shou Feng

**Affiliations:** Obstetrical Department, Changzhou Women and Children Health Hospital Affiliated to Nanjing Medical University, Changzhou, Jiangsu China

**Keywords:** Foley catheter balloon, Induction of labor, Intrauterine infection, Bishop score, Cervical ripening

## Abstract

**Background:**

Previous studies had found that the mechanical methods were as effective as pharmacological methods in achieving vaginal delivery. However, whether balloon catheter induction is suitable for women with severe cervical immaturity and whether it will increase the related risks still need to be further explored.

**Research aim:**

To evaluate the efficacy and safety of Foley catheter balloon for labor induction at term in primiparas with different cervical scores.

**Methods:**

A total of 688 primiparas who received cervical ripening with a Foley catheter balloon were recruited in this study. They were divided into 2 groups: Group 1 (Bishop score ≤ 3) and Group 2 (3 < Bishop score < 7). Detailed medical data before and after using of balloon were faithfully recorded.

**Results:**

The cervical Bishop scores of the two groups after catheter placement were all significantly higher than those before (Group 1: 5.49 ± 1.31 VS 2.83 ± 0.39, *P*<0.05; Group 2: 6.09 ± 1.00 VS 4.45 ± 0.59, *P*<0.05). The success rate of labor induction in group 2 was higher than that in group 1 (*P*<0.05). The incidence of intrauterine infection in Group 1 was higher than that in Group 2 (18.3% VS 11.3%, *P*<0.05).

**Conclusion:**

The success rates of induction of labor by Foley catheter balloon were different in primiparas with different cervical conditions, the failure rate of induction of labor and the incidence of intrauterine infection were higher in primiparas with severe cervical immaturity.

**Supplementary Information:**

The online version contains supplementary material available at 10.1186/s12884-024-06600-1.

## Background

At present, the most commonly used methods to promote cervical ripening are pharmacological (prostaglandins or isosorbide mononitrate) and mechanical methods.

(insertion of balloons catheter or cervical dilators [1–5]. Mechanical methods have been recommended by many institutions, such as the ACOG [6], WHO [7] and Canada IOL guide lines [8]. Previous studies have found that the mechanical methods were as effective as pharmacological methods in achieving vaginal delivery [3, 9–13].

However, there is still less evidence on whether women with severe cervical immaturity are suitable for promoting cervical ripening by mechanical methods. And whether intrauterine placement of “foreign bodies” will additionally increase the risk of infection, and most of the current articles do not mention the risk of infection at all [14, 15], so further research is needed. The purpose of this study was to evaluate the efficacy and safety of the use of Foley catheter balloon for labor induction at term in primiparas under different cervical conditions.

## Methods

### Design

A retrospective study was undertaken between January,2018 and December, 2022 at Changzhou Women and Children Health Hospital affiliated to Nanjing Medical University. All patients signed informed consents, the study design and protocol were reviewed and approved by the Institutional Review Board of Changzhou Women and Children Health Hospital affiliated to Nanjing Medical University prior to the initiation of the study in October 25, 2017.

### Sample

From January, 2018 to December, 2022, 688 primiparas who intended to receive cervical ripening with a Foley catheter balloon with a clinical need were recruited. Eligibility criteria included: term (37–42 weeks), singleton, primipara, intact membranes, Bishop score of < 7, cephalic presentation and appropriately grown; cervical ripening being done for such reasons: at or beyond 41 weeks, gestational hypertension, gestational diabetes, oligoamnios and other maternal factors, excluding postdates and social inductions. Women were excluded if they had placenta praevia or any other contraindication to vaginal delivery. According to the cervical Bishop scores, the patients were divided into two groups: Group 1 (Group of severe cervical immaturity, Bishop scores ≤ 3) and Group 2 (Group of cervical immaturity, 3 < Bishop scores < 7).

Before the starting of the ripening and induction process, the Bishop score was determined by a fixed, experienced, senior physician for each treatment group by digital examination. A standardised Bishop score was used for this purpose [16], scoring was as follows: Position of cervix (posterior: 0, intermediate: 1, anterior: 2), consistency of the cervix (firm: 0, intermediate: 1, soft: 2), effacement (0–30%:0, 31–50%: 1, 51–80%: 2, > 80%: 3), dilation (0 cm: 0, 1–2 cm: 1, 3–4 cm: 2, > 5 cm: 3), fetal station (− 3: 0, − 2: 1, − 1 and 0: 2, + 1 and + 2: 3), with a maximum score of 13.

The Foley catheter balloons were inserted by specially trained doctors. On the first day, because the catheter was to be removed after 12 h of placement, we chose to insert the Foley catheter balloon at 8 pm in order to facilitate catheter removal and subsequent processing procedures during working hours the next day. Accordance to the manufacturer’s instructions, after strict disinfection, without touching the vaginal wall, the Foley catheter balloon was placed through the cervical canal to the internal cervical opening with the aid of a sterile speculum., filled with 80 mL normal saline, and then pulled close to the internal cervical opening. The Foley ball catheter was fixed by tape on the inner thigh of the patient under gentle traction. The Foley catheter balloon was removed if the following conditions occurred: (1) spontaneous rupture of membranes; (2) regular uterine contractions; (3) signs of intrauterine infection; (4) uterine hyperstimulation; (5) fetal distress; (6) abnormal bleeding suspicious for placental abruption.

If the Foley catheter balloon did not fall out spontaneously, it was removed after 12 h. After removal of the balloon catheter, the Bishop scores were assessed again, and fetal heart rate and uterine contractions were observed for an hour. Further management of labor was expectant management (if regular uterine contractions are present), amniotomy and/or intravenous (IV) oxytocin (if regular uterine contractions are not present, i.e., < 3 contractions/10 min). And the timing of artificial rupture of membranes was decided by the assessment of obstetricians. Oxytocin was administered intravenously at a concentration of 0.5% oxytocin was given intravenously 8 drops per minute (8 drops/min), The fetal heart rate was monitored and the titer of oxytocin was increased by 8 drops/min every 20 min or more until regular uterine contractions occurred. The maximum titer of oxytocin was 40 drops/min, and the maximum concentration was 1%. If labor was not initiated at 5 pm, oxytocin administration was stopped and performed again at 8 am on the following days until delivery. The choice of induction of labor would be based on clinical risk assessment by the treating clinicians according to the hospital protocol. Electronic fetal heart monitoring was performed for all patients.

### Data collection

We faithfully recorded the detailed medical data, including maternal ages (years), gestational ages, Bishop scores (before and 12 h after catheter placement), methods of delivery (spontaneous vaginal delivery, instrumental, caesarean section), indications for caesarean section (intrauterine infection, lack of progress, meconium-stained amniotic fluid, fetal distress, failed induction of labor and social factor), the mean Foley balloon catheter insertion to delivery intervals, and labor complications (postpartum hemorrhage, fetal distress, intrauterine infection, hyperstimulation, incomplete rupture of uterus, placental abruption and perineum hematoma). All the placentas were pathologically inspected, intrauterine infection was diagnosed as maternal fever (≥ 38 °C), accompanied by maternal tachycardia (> 100 bpm), or uterine fundal tenderness, or fetal tachycardia (> 160 bpm), or purulent amniotic fluid, or chorioamnionitis found by placental pathologic examination.

### Data analysis

All statistical analysis were performed using SPSS 10.0, Quantitative data were expressed as Mean ± SD, the difference of quantitative data between the two groups were analyzed by homogeneity test of variances, and t test was used under an equal condition, t ‘test should be carried out if the variance of the two groups is not equal. The enumeration data were expressed as rate (%), the chi-square tests were used for comparison between the two groups. *P* < 0.05 was chosen to be statistically significant.

## Results

**Fig. 1:**
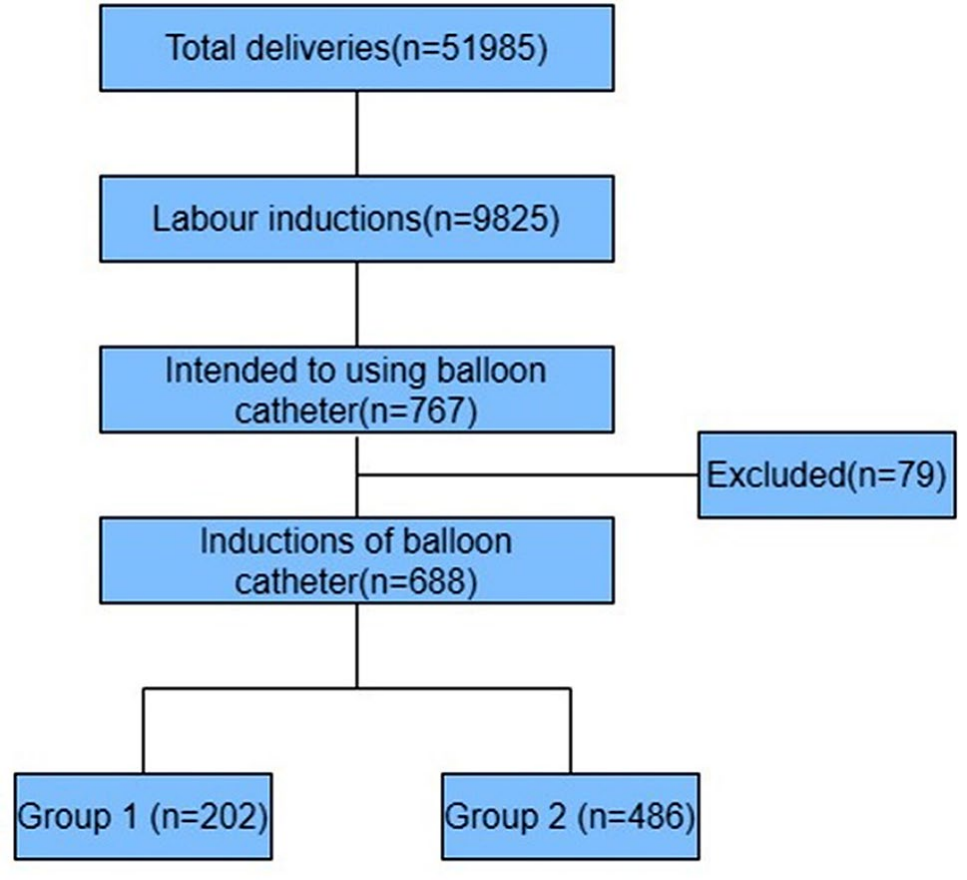
The trial profile

The trial was carried out from January, 2018 to December, 2022. During this study period, 51,985 women delivered in our hospital, and labor was induced in 9825 (18.9%). A total of 767 primiparas intended to receive cervical ripening with a Foley catheter balloon, who were hospitalized in the ward of Changzhou Women and Children Health Hospital, but 79 cases gave up using Foley catheter balloon because of vaginal inflammation or severe cervical erosion, and finally 688 cases successfully received cervical ripening with a Foley catheter balloon, including 202 cases in Group 1 and 486 cases in Group 2. No women were excluded from the study due to catheter associated issues: clinician unable to insert catheter, spontaneous rupture of membranes on balloon inflation and fetal heart rate deceleration noted at insertion. The trial profile was shown in Fig. 1.

The baseline demographic characteristics and the clinical features were shown in Table 1, There was no significant difference in related indicators between the two groups (*P* > 0.05). The indications for induction of labor were given in Table 2, most of the inductions in this study were all carried out because the pregnancies were at or beyond 41 weeks of gestation (50.0%, 49.6%), and there was no difference in indications between the two groups (*P* > 0.05).

**Table 1:** Baseline main demographic and clinic characteristics

Variables	Group 1 *n*=202	Group 2 *n*=486	*P* value
Maternal age (years)	27.93±3.68(19-43)	27.94±3.52(16-39)	>0.05^1^
Gestational age (weeks)	39.88±1.00(37-41)	39.95±0.91(37-41)	>0.05^1^
BMI (kg/m2)	26.51±3.72(18.14-40.01)	26.47±3.68(18.07-39.26)	>0.05^1^
Pregnancy	1.63±0.35(1-4)	1.59±0.37(1-3)	>0.05^1^
Birth weight (g)	3473.17±412.87(2540-4680)	3433.35±377.60(2100-4750)	>0.05^1^
Epidural analgesia	128(63.4%)	354(72.8%)	>0.05^2^

Quantitative data were expressed as Mean ± SD (range), enumeration data were expressed as rate (%); 1 t test. 2Chi-square. *P* < 0.05 was considered significant

**Table 2:** The indications for induction of labor

Indication	Group 1 *n* =202(%)	Group 2 *n* =486(%)	*P* value
≥ 41 weeks	101(50.0)	241(49.6)	0.933^1^
Gestational diabetes	40 (19.8)	96(19.8)	1.00^1^
Gestational hypertension	22 (10.9)	58(11.9)	0.794^1^
Oligoamnios	21 (10.4)	53(10.9)	0.893^1^
Other	18 (8.9)	38(7.8)	0.647^1^

Data are absolute and relative frequencies. ^1^Chi-square

The cervical Bishop scores of the two groups after catheter placement were all significantly higher than those before (Group 1: 5.49 ± 1.31 VS 2.83 ± 0.39, *P*<0.05; Group 2: 6.09 ± 1.00 VS 4.45 ± 0.59, *P*<0.05) (Table 3). The mean balloon catheter insertion to delivery intervals were significantly different between the two groups (32.55 ± 9.56 VS 29.87 ± 10.01 h, *P*<0.05) (Table 4).

**Table 3:** Changes in cervical Bishop score

Bishop scores	Group 1 *n* =202	Group 2 *n* =486
before catheter placement	2.83±0.39(1-3)	4.45±0.59(4-6)
after catheter placement	5.49±1.31(3-8)	6.09±1.00(4-8)
*P* value	<0.05	<0.05

Data was shown as Mean ± SD (range); *P* < 0.05 was considered significant

The outcome parameters were shown in Table 4. In the Group 1, 13 cases (6.4%) failed to induce labor, and the success rate of induction was 93.6%. In the Group 2, 13 cases (2.7%) failed to induce labor, and the success rate of induction was 97.3%. The success rate of labor induction in Group 2 was higher than that in Group 1, and the difference was statistically significant (*P*<0.05). But there was no significant difference in the methods of delivery between the two groups (*P* > 0.05). The incidence of intrauterine infection in Group 1 was higher than that in Group 2 (*P*<0.05). The incidences of postpartum hemorrhage and fetal distress did not differ significantly between the two groups (*P* > 0.05).

**Table 4:** The outcome parameters

outcome parameters	Group 1 *n*=202 (%)	Group 2 *n*=486(%)	*P* value
**Induction of labor**			
Successful induction	189 (93.6)	473(97.3)	0.027^1^
Failed induction	13 (6.4)	13 (2.7)	
**The methods of delivery**			
Spontaneous vaginal delivery	139 (68.8)	358 (73.7)	0.234^1^
Instrumental	2 (2.0)	9 (1.9)	
Caesarean section	61 (30.2)	119 (24.5)	
**The mean intervals**	32.55±9.56(23-34)	29.87±10.01(19-31)	0.0013^2^
**Labor complications**			
Intrauterine infection	37 (18.3)	55 (11.3)	0.019^1^
Postpartum hemorrhage	17 (8.4)	39 (8.0)	0.879^1^
Fetal distress	22 (10.9)	42 (8.6)	0.388^1^
Incomplete rupture of uterus	1(0.5)	0(0)	
Placental abruption	0(0)	1(0.2)	
Perineum hematoma	0(0)	1(0.2)	
Prolapse of cord	0(0)	1(0.2)	
Hyperstimulation	0(0)	0(0)	

Quantitative data were expressed as Mean ± SD (range), enumeration data were expressed as rate (%); ^1^Chi-square, ^2^ t test

The indications for caesarean section were shown in Table 5. The rate of cesarean section due to intrauterine infection was significantly different between the two groups (*P*<0.05), and there were no significant difference between the two groups in the other indications for cesarean section (*P* > 0.05).

**Table 5:** The indications for caesarean section

The indications	Group 1 *n*=61(%)	Group 2 *n*=119 (%)	*P* value
Intrauterine infection	12 (19.7)	41 (34.5)	0.346^1^
Lack of progress	13 (21.3)	31 (26.0)	1.00^1^
Fetal distress	12 (19.7)	18 (15.1)	0.219^1^
Meconium-stained amniotic fluid	10 (16.4)	14 (11.8)	0.178^1^
Failed induction of labor	13 (21.3)	13 (10.9)	0.027^1^
Other factor	1 (1.6)	2(1.7)	1.00^1^

Data were expressed as rate (%); ^1^Chi-square

## Discussion

Induction of labor (IOL) is one of the most common interventions among pregnant women. Most recently in the United States, the rate of induction was approximate 23.3% [17]. In our hospital, the rate of induction was 18.9%. 49.7% of the inductions were carried out because the pregnancies were at or beyond 41 weeks of gestation, gestational diabetes (19.8%) and hydramnios (11.6%) were also important indications of labor induction, which was basically consistent with what S Kehl et al. reported [18]. In Germany, the failure of catheter placement occurred in 4 out of 168 women [18]. But in our study, all the Foley catheter balloons were placed successfully, it may be related to the fact that all our doctors who were responsible for catheter placement have been specially trained.

Previous literature suggested that balloon catheter could significantly improve cervical scores [19–24]. In our study, it was also found that Foley catheter balloons were very effective in improving cervical scores, and had obvious effects on pregnant women with different cervical conditions. Similarly, 28 trials (6619 women) showed that mechanical induction with a balloon is as effective as vaginal PGE2 [25]. The Foley catheter can produce mechanical dilatation of the cervical canal after placement. At the same time, The Foley catheter can also cause the stretch of fetal membranes and cervical cells after correct positioning, leading to increased secretion of prostaglandin E2 (PGE2) and interleukin-8 (IL-8), which are key mediators of cervical maturation. PGE2 also has the ability to directly stimulate uterine contractions. Additionally, cyclic mechanical stretching can greatly increase collagenase activity and hyaluronic acid expression in fibroblasts (which concentration greatly increases in cervical tissue at term) which enhances the influx of water to the cervical stroma leading to collagen fibers reorganization [26–29].

In our study, the overall success rate of induction of labor was more than 96%, this was basically consistent with Wen C ‘s study [30] (97.8%). In a meta-analysis study [31], it was found that the total vaginal delivery rate of single balloon catheter was 78.8%. While in our study, the overall vaginal delivery rate was 73.8%, which was slightly lower than in the previous study. However, most studies did not mention the effect of different cervical scores on catheter balloon induction of labor. In Wen C ‘s study [30], it was only found that patients with initial Bishop score ≤ 3 and patients with initial Bishop score of 4–6 had significant improvement in Bishop score after balloon promotion of cervical ripening, but the influence of other aspects was not mentioned. In our study, we found that there was no significant difference in the final methods of delivery between the two groups. However, the success rate of induction of labor by Foley balloon catheter was different in pregnant women with different cervical conditions. The success rate of induction of labor by Foley balloon catheter was lower in primiparas with severe cervical immaturity, suggesting that whether to choose balloon catheter or other methods of induction of labor needs further consideration for primiparas with severe cervical immaturity.

In this study, we focused on the risks associated with Foley balloon catheter labor induction in primiparas with different cervical conditions. We found that there were no significant differences in the incidences of postpartum hemorrhage and fetal distress between the two groups. Most researchers believe that balloon catheter does not significantly increase the risk of delivery [32, 33], the 2023 literature review [25] similarly supports this view.

It is still controversial whether the placement of a “foreign body” into the uterus increases the risk of intrauterine infection or not, the evidence being astoundingly sparse and contradictory. Most studies did not report on this outcome, resulting in limited data [25]. Among all primiparas in our study, we found that the overall incidence of intrauterine infection was 13.4%, the meta-analysis by Heinemann [34] that included 30 randomised controlled trials (*n* = 4468) comparing the Foley catheter to medical methods of induction similarly found a significantly increased rate of maternal infections (7.6 vs. 5.0 %, pooled OR: 1.50; 95 % CI 1.07–2.09). In the study by Yan J et al. [13], it was similarly found that induction of labor by mechanical catheter was associated with a higher incidence of chorioamnionitis compared with medical induction. And we also found that the incidence of intrauterine infection was higher in primiparas with severe cervical immaturity, which we analyzed as a result of severe cervical immaturity leading to a longer interval and, ultimately, a higher incidence of intrauterine infection. Therefore, careful evaluation of cervical conditions and minimizing the interval between balloon insertion and delivery may reduce the incidence of intrauterine infection.

It must be recognized that the Bishop method for cervical assessment is a somewhat subjective index, which has a certain disadvantage of non-repeatability. In our study, we have taken some measures to make up for these shortcomings as far as possible. For example: the Bishop score was determined by a fixed, experienced, senior physician for each treatment group by digital examination; Cervical maturity was assessed by the same medical staff before and after induction of labor. Moreover, vaginal ultrasound can be used to evaluate the cervical conditions to improve the predictive value [35–37].

## Conclusion

The success rates of induction of labor by Foley catheter balloon were different in primiparas with different cervical conditions, the failure rate of induction of labor and the incidence of intrauterine infection were higher in primiparas with severe cervical immaturity.

### Electronic supplementary material

Below is the link to the electronic supplementary material.

Supplementary Material 1

Supplementary Material 2

## Data Availability

Data is provided within the manuscript or supplementary information files.
